# Durable recovery from amblyopia with donepezil

**DOI:** 10.1038/s41598-023-34891-5

**Published:** 2023-06-22

**Authors:** Carolyn Wu, Eric D. Gaier, Bharti R. Nihalani, Sarah Whitecross, Takao K. Hensch, David G. Hunter

**Affiliations:** 1 Department of Ophthalmology, Boston Children’s Hospital, Boston, MA USA; 2 Department of Ophthalmology, Harvard Medical School, Boston, MA USA; 3 Picower Institute for Learning and Memory, Massachusetts Institute of Technology, Cambridge, MA USA; 4 Center for Brain Science, Department of Molecular Cellular Biology, Harvard University, Cambridge, MA USA; 5 International Research Center for Neurointelligence, University of Tokyo Institutes for Advanced Study, Tokyo, Japan; 6 FM Kirby Neurobiology Center, Boston Children’s Hospital, Harvard Medical School, Boston, MA USA

**Keywords:** Eye diseases, Translational research

## Abstract

An elevated threshold for neuroplasticity limits visual gains with treatment of residual amblyopia in older children and adults. Acetylcholinesterase inhibitors (AChEI) can enable visual neuroplasticity and promote recovery from amblyopia in adult mice. Motivated by these promising findings, we sought to determine whether donepezil, a commercially available AChEI, can enable recovery in older children and adults with residual amblyopia. In this open-label pilot efficacy study, 16 participants (mean age 16 years; range 9–37 years) with residual anisometropic and/or strabismic amblyopia were treated with daily oral donepezil for 12 weeks. Donepezil dosage was started at 2.5 or 5.0 mg based on age and increased by 2.5 mg if the amblyopic eye visual acuity did not improve by 1 line from the visit 4 weeks prior for a maximum dosage of 7.5 or 10 mg. Participants < 18 years of age further patched the dominant eye. The primary outcome was visual acuity in the amblyopic eye at 22 weeks, 10 weeks after treatment was discontinued. Mean amblyopic eye visual acuity improved 1.2 lines (range 0.0–3.0), and 4/16 (25%) improved by ≥ 2 lines after 12 weeks of treatment. Gains were maintained 10 weeks after cessation of donepezil and were similar for children and adults. Adverse events were mild and self-limited. Residual amblyopia improves in older children and adults treated with donepezil, supporting the concept that the critical window of visual cortical plasticity can be pharmacologically manipulated to treat amblyopia. Placebo-controlled studies are needed.

## Introduction

Amblyopia is a developmental brain disorder caused by abnormal visual input early in life that results in decreased vision in an otherwise structurally normal eye. With a prevalence of 2–3%^1–3^, amblyopia is the leading cause of monocular visual impairment in children and adults. Occlusion (patching) and pharmacological penalization (via atropine) are conventional treatment modalities for amblyopia during the critical period of visual development. While amblyopia may be reversible when therapy is initiated during this period, delayed treatment in older children and adults is less effective due to loss of brain plasticity^4^. In one study among 507 children aged 7 to 17 years, only 25% more children benefitted from additional amblyopia treatment compared to optical correction alone^5^. Given the risks and lasting disadvantage of reduced binocular vision, alternative approaches to treat residual amblyopia in older children and adults are urgently needed.

Visual experience affects the development of the visual pathways in early infancy and childhood during a time termed the “critical period.” Animal models have revealed the molecular mechanisms that determine critical period closure^6^. These ‘brakes’ serve to limit neuroplasticity and recovery from amblyopia. Key effectors of critical period closure include transition from excitation to inhibition and establishment of structural barriers to plasticity including perineuronal nets^7^. Novel strategies to restore a juvenile-like state permissive of neuroplasticity should therefore lift the brakes on critical period closure for successful amblyopia treatment later in life.

Lynx1 is one such endogenous protein ‘brake’ expressed in the primary visual cortex that negatively modulates nicotinic cholinergic signaling. Its expression increases with critical period closure across cortical areas^8^, and in mice lacking Lynx1, critical period plasticity fails to close. Notably, such extended visual cortical plasticity and susceptibility to amblyopia after the age at which the critical period normally closes is dependent on cholinergic signaling^9^. Genetic deletion of Lynx1 permits recovery of visual function with reverse occlusion in amblyopic adult mice. Similar effects are seen in wildtype mice systemically treated with physostigmine, an acetylcholinesterase inhibitor (AChEI) to reverse amblyopia in adulthood^9^. Several studies have also shown that cholinergic enhancement in healthy human adults augments neural plasticity and can increase the magnitude and specificity of visual perceptual learning^10–14^. Here, we leveraged this translational opportunity to the clinic.

Donepezil is an AChEI with a well-defined, favorable safety profile that is FDA-approved for the use of cognitive impairment associated with Alzheimer dementia. Given the promising preclinical data in mice, we hypothesized that reactivation of plasticity after critical period closure using donepezil could enable recovery from amblyopia^15^. To test this hypothesis, we executed an open-label pilot study using oral donepezil to treat residual amblyopia in children and adults ≥ 8 years of age.

## Methods

**Figure 1:**
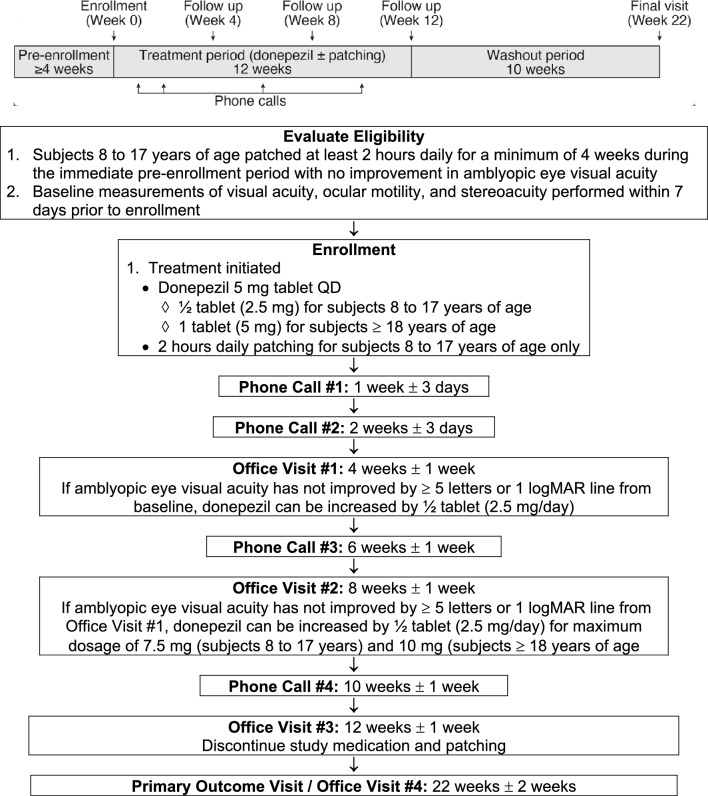
Study design flowchart.

The study was approved by the Institutional Review Board at Boston Children’s Hospital. All research was performed in accordance with the ethical standards of Boston Children’s Hospital and with the 1964 Declaration of Helsinki and its later amendments. Written informed consent was obtained from adult subjects or parents/guardians of minor subjects. Written assent was obtained from appropriately-aged subjects when applicable. Approval of an investigational new drug (IND) application was obtained from the Food and Drug Administration (FDA) for the use of donepezil to treat amblyopia. The study is registered at ClinicalTrials.gov (identifier NCT01584076; first posted 24/04/2012). The study design is summarized in Fig. 1.

### Subjects

This was an open-label pilot study of donepezil efficacy in amblyopia treatment. We sought to only enroll subjects whose amblyopia treatment had been or was being discontinued given no further improvement in amblyopic eye visual acuity. All subjects must have undergone prior amblyopia treatment with patching. Additional major eligibility criteria included age ≥ 8 years, best corrected visual acuity (BCVA) in the amblyopic eye 20/50-20/400, BCVA in the sound eye of ≥ 20/25, and presence or history of strabismus and/or anisometropia sufficient to cause amblyopia. At the time of enrollment, subjects must have been wearing optimal optical correction for a minimum of 4 weeks with stable amblyopic eye visual acuity (< 5 letters or 1 logMAR line of improvement during 2 consecutive visual acuity measurements). Subjects 8 to 17 years of age underwent a run-in period of amblyopia treatment consisting of at least 2 h of daily patching for at least 4 weeks during the immediate pre-enrollment period, with no improvement in best-corrected amblyopic eye visual acuity (< 5 letters or 1 logMAR line of improvement during 2 consecutive visual acuity measurements at least 4 weeks part) to ensure subjects would not improve with additional patching alone given the past extensive history of amblyopia treatment. Subjects ≥ 18 years of age were required to have a history of prior patching treatment for amblyopia.

Major ophthalmic exclusion criteria included deprivation amblyopia, myopia < − 6.00 D spherical equivalent, presence of associated findings that could cause reduced visual acuity, previous intraocular or refractive surgery, strabismus surgery planned within 22 weeks, current vision therapy or orthoptics, and treatment with topical atropine up to 4 weeks prior to enrollment. Systemic exclusion criteria included presence of cardiac condition, asthma, obstructive pulmonary disease, seizure disorder, urinary incontinence, peptic ulcer disease receiving concurrent NSAIDs, history of gastrointestinal bleeding from peptic ulcer disease, known psychological problems, prior AChEI treatment, current use of medication for the treatment of ADHD or psychological disorders, and females who were pregnant or lactating or who intended to become pregnant within the following 6 months.

### Treatment

The study intervention consisted of daily oral donepezil with a starting dosage of 2.5 mg for subjects aged 8 to 17 years and 5.0 mg for subjects aged ≥ 18 years. The dosage of donepezil was increased by 2.5 mg if visual acuity did not improve by 5 letters or 1 logMAR line from the visit 4 weeks prior, with a maximum dosage of 7.5 mg for subjects aged 8 to 17 years and 10 mg for subjects aged ≥ 18 years. Subjects aged 8 to 17 years also continued 2 h of daily patching. Animal models showed improvements in amblyopic eye visual acuity with administration of acetylcholinesterase inhibitors alone. Despite understanding that patching would add a confound, we found it necessary to implement this to the design because we (1) did not want to withhold patching from the younger age group, and (2) knew it would be even more difficult to enroll adults in the older age group if they were required to patch. Previous studies comparing near versus distance activities while patching found no difference in amblyopic eye visual acuity outcomes^16,17^. Subjects were not instructed to perform specific activities during patching. A calendar log indicating hours per day patched was completed by each subject. Patching compliance (hours patched/hours patching prescribed) was evaluated by the calendar log. Medication compliance (tablets taken/tablets prescribed) was evaluated by counting tablets in the returned medication bottles.

### Ophthalmic testing

Visual acuity was measured (with best correction in spectacles where indicated) using the Electronic Early Treatment of Diabetic Retinopathy Study (E-ETDRS) method. Stereoacuity was measured with the Randot Stereotest (Stereo Optical Co, Inc. Chicago, IL). All stereopsis thresholds were converted to log arcsec for mathematical analysis. Stereoacuity threshold was set at 10,000 arcsec for patients who did not demonstrate stereopsis and were unable to perceive depth in the Stereo Fly test (Stereo Optical, Inc.), 3000 arcsec stimulus^18^.

### Follow-up

Follow-up office visits occurred at weeks 4, 8, 12, and 22, with each visit required within ± 1 week of the target date. Treatment (donepezil ± patching) was continued for the first 12 weeks and discontinued thereafter. The final primary outcome visit was at 22 weeks, 10 weeks after donepezil ± patching were discontinued.

Adverse effects were assessed at each office visit and during phone calls conducted 1, 2, 6, and 10 weeks after enrollment. All adverse effects were recorded even if considered unrelated to the study treatment.

### Statistical analyses

All participants completed the study, so no adjustments for missing data were needed. Descriptive statistics (mean, standard deviation) were calculated using Microsoft Excel (version 16.50), and all other statistical tests were performed using Prism (Version 9.1.2 for macOS, GraphPad Software, LLC.). Changes in BCVA and stereoacuity were assessed using a Wilcoxon signed-rank test. (The Shapiro–Wilk test for normality was significant for the amblyopic eye BCVA letters in the < 18-year-old group, thus the data are not normally distributed, necessitating a non-parametric statistical approach). Comparisons of treatment responses (change in BCVA) between subgroups employed the Mann–Whitney test of ranks. In all cases, two-tailed *p* values < 0.05 were considered statistically significant.

## Results

**Figure 2:**
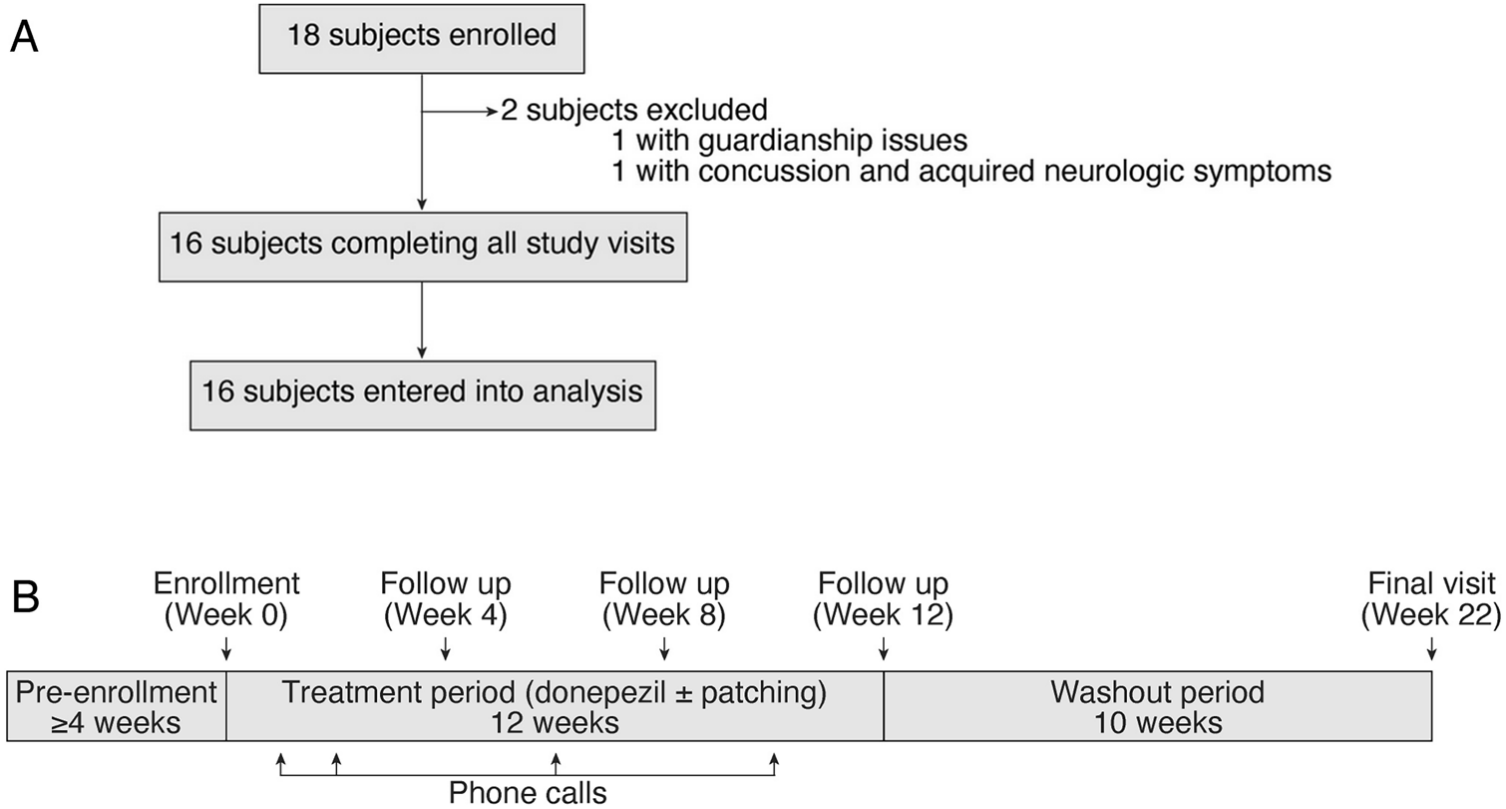
Subject enrollment.

Eighteen subjects were enrolled in the study (Fig. 2). Two subjects were excluded; one due to guardianship issues and the other due to a concussion sustained after enrollment with persisting neurological symptoms. Table 1 lists the baseline characteristics of the remaining 16 subjects in the study group. Mean age was 16 years (range 9–37) with 11 in the 8 to 17 age group and 5 in the ≥ 18 age group (ages 18, 18, 23, 27, and 37 years). Six (38%) were female and 9 (56%) were white. Amblyopic eye BCVA ranged from 20/63-20/250 with a mean of 20/140 and median of 20/125. Mean amblyopic eye BCVA was 20/125 (range 20/63-20/250) in the 8 to 17 age group and 20/160 (range 20/63–20/250) in the ≥ 18 age group. Subjects in the 8- to 17-year-old group had undergone previous amblyopia treatment for a mean of 30 months (range 8–60) that had been or was being discontinued prior to enrollment given no further improvement in amblyopic eye acuity. In addition to patching, 9 subjects were also previously treated with atropine and 1 subject with a Bangerter foil. No potential subjects showed improvement in amblyopic eye visual acuity during the 4-week patching period required for enrollment.

**Table 1:** Baseline demographic and clinical characteristics.

	N = 16	%
Female	6	38
Race or ethnicity		
Asian	0	0
Black/African American	1	6
Hispanic/Latino	4	25
White	9	56
Mixed	2	13
Age		
8 to 17 years	11	69
≥ 18 years	5	31
Mean (years)	16	
Cause of amblyopia		
Strabismic	1	6
Anisometropic	5	31
Mixed	10	63
Amblyopic eye visual acuity		
≤ 20/250 (≤ 33 letters)	4	25
20/100–20/200 (34–53 letters)	8	50
20/80 (54–58 letters)	1	6
20/63 (59–63 letters)	3	19
Mean	20/125	

All 16 subjects completed all study visits, though 6 subjects had study visits conducted outside of the specified study window due to scheduling, concerns for in-office visits during the COVID–19 pandemic, or inclement weather. Treatment duration was consistent with the study protocol and adherence to prescribed medication and occlusion therapies were > 95% on average (Table 2).

**Table 2:** Study treatment.

	Mean (range)
Time on treatment	13 weeks (11 1/7–15 weeks)
Time off treatment to primary outcome visit	10 5/7 weeks (7 5/7–15 weeks)
Compliance with medication	97% (88–100%)
Compliance with patching	95% (74–100%)

**Figure 3:**
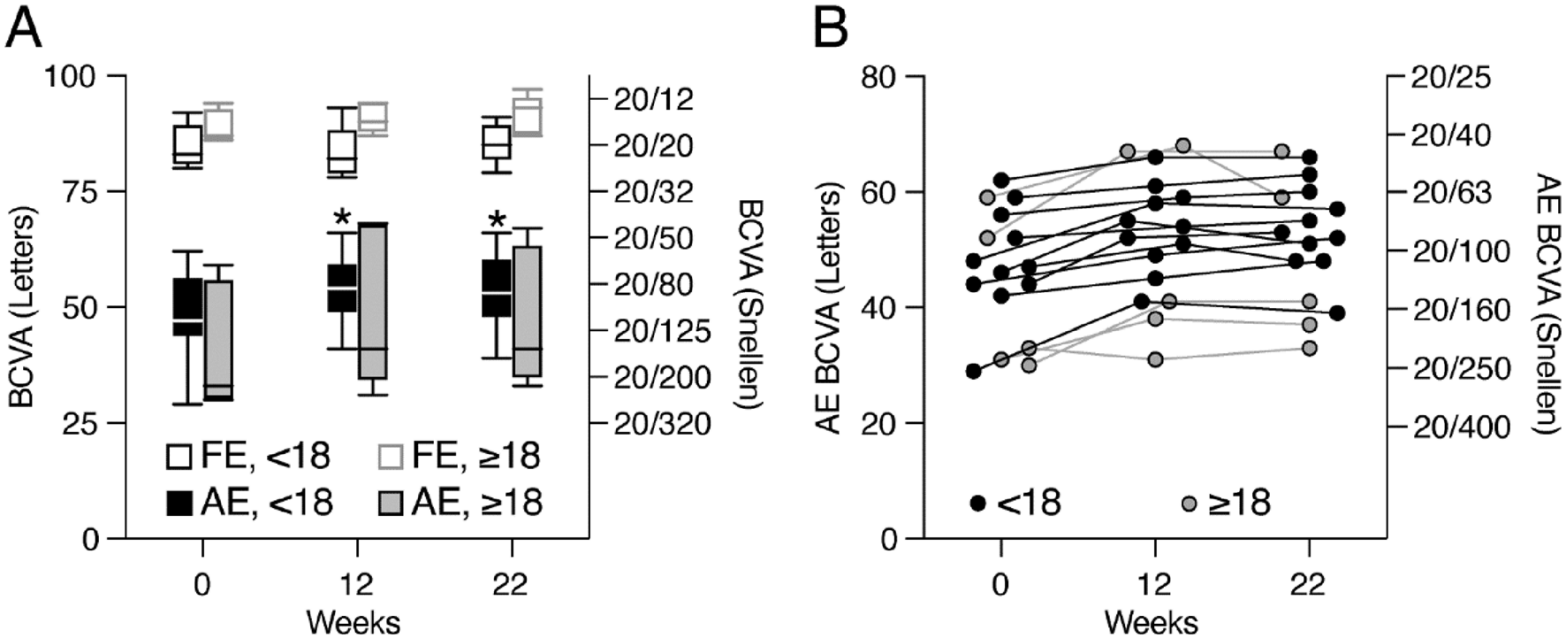
Improvement in best corrected visual acuity by age. (**A**) Best corrected visual acuity (BCVA) of amblyopia eyes (AE; filled) and fellow eyes (FE; open) separated by younger (< 18 years; black) and older (≥ 18 years; gray) age groups plotted for each visit. Whiskers depict the minimum–maximum, boxes depict the quartile boundaries, and lines depict medians. *denotes p < 0.05 compared to week 0 (Wilcoxon signed rank test). (**B**) BCVA of AE for individual subjects at each visit.

Median improvement in amblyopic eye BCVA from baseline to 12 weeks of treatment was 1.2 (97.87% CI 0.6–2.0) lines (Fig. 3). This improvement was sustained at the final outcome visit at 22 weeks, 10 weeks after stopping treatment (median improvement 1.0 lines (97.87% CI 0.6–1.8)). Improvement of ≥ 2.0 lines (≥ 10 ETDRS letters) was noted in 25% (4/16) of subjects.

At 12 weeks, amblyopic eye BCVA improvement was similar between age groups (< 18: median 0.8 lines, 95% CI 0.70–1.51 lines; ≥ 18: median 1.2 lines, 95% CI − 0.37–2.93 lines; *p* = 0.9). The response appeared similar regardless of the presence of strabismus, with the number of subjects too small for meaningful statistical analysis (No strabismus: median 0.7 lines, 95% CI − 0.15–1.45 lines, n = 4; Strabismus: median 1.4 lines, 95% CI 0.77–1.90 lines, n = 12; *p* = 0.18). Mean baseline BCVA of the fellow eye was 20/20 (86 letters) (range 20/12–20/25) and was similar between age groups (< 18: 20/20, range 20/16–20/25; ≥ 18: 20/16, range 20/12–20/20). Fellow eye BCVA did not change from baseline to 12 weeks of treatment (median change: − 0.2 lines; 97.87% CI − 0.4–0.2 lines, *p* > 0.2]) or to the final visit (median change: − 0.2 lines; 97.87% CI − 0.4–0.6 letters, *p* > 0.7]).

Mean stereoacuity at baseline was 3.5 log arcsec (range 1.3–4.0 log arcsec) and was similar between age groups (< 18: 3.5 log arcsec, range 1.3–4.0; ≥ 18: 3.5 log arcsec, range 1.8–4.0). Stereoacuity did not change from baseline to 12 weeks of treatment (median change: 0.0 log arcsec [97.87% CI − 0.5–0.3 log arcsec, *p* > 0.9]) or to the final visit (median change: 0.0 log arcsec [97.87% CI − 0.5–0.0 log arcsec, *p* > 0.15]). One the 6 patients with measurable stereoacuity at baseline and two patients without measurable stereopsis at baseline showed improvement at the 12- and 22-week follow-up visits (Supplementary Fig. 1).

All adverse effects were mild and self-limited (Table 3). The most common complaints were gastrointestinal disturbances, headache, fatigue, and lightheadedness/dizziness. The donepezil dose was held or lowered in three subjects due to adverse effects, but all were able to achieve the protocol-determined donepezil dosage by increasing over a longer time interval. Donepezil was not discontinued for any patient during the study.

**Table 3:** Adverse effects.

Adverse effect	#Patients
Loss of appetite	4
Nausea	4
Headache	3
Tired/sleepy	3
Lightheaded/dizziness	3
Stomach cramps/pain	3
Diarrhea	3
Vomiting	3
Anxious/nervous	2
Difficulty falling asleep	2
Muscle cramps	2
Accident with stools	1
Change in color of stools	1

## Discussion

In this open-label pilot study, we gathered helpful data that informs the feasibility of a larger randomized trial that would be required to demonstrate efficacy. We established precedent in administering donepezil in the context of amblyopia and evaluated key factors including an escalating dosing regimen, adverse effects, and recruitment potential. Donepezil (± patching) treatment improved amblyopic eye visual acuity by 1.2 lines, and 25% of subjects improved by ≥ 2 lines after only 12 weeks of treatment. Most notably, gains in visual acuity were sustained 10 weeks after cessation of treatment.

The impact of donepezil treatment on stereoacuity was not clear. Two subjects with anisometropic amblyopia without baseline stereopsis developed stereopsis during the study (nil to 3000 arcsec with 2.0 lines improvement in amblyopic eye BCVA; nil to 200 arcsec with 1.8 lines improvement in amblyopic eye BCVA). One subject whose amblyopic eye BCVA improved 2.2 lines maintained no stereopsis throughout the study; however, stereopsis improved to 200 arcsec following strabismus surgery performed after the study. While only 1/6 patients with demonstrable stereoacuity at baseline improved during the study, 4 of those 6 patients had less than 1 line improvement in amblyopic eye BCVA. Overall, there was a lack of general effect, but this may have been contributed to in part by the high proportion of subjects with strabismus and the small sample of subjects with measurable stereoacuity at enrollment. As such, we cannot conclude that stereoacuity is uninfluenced by donepezil treatment of amblyopia.

Adherence with treatment was very high for both the medication and patching. The treatment was well tolerated overall, with no serious adverse effects and no subjects discontinued the study due to adverse effects. Donepezil has an established safety profile in clinical trials to improve cognitive and behavioral function in children ≥ 2 years of age with fragile X syndrome, autism spectrum disorder, Down syndrome, and attention deficit hyperactivity disorder^19–31^. Therefore, our results are consistent with those obtained in other contexts and in younger children, suggesting that adverse risks of donepezil use are low and favor further study in the context of amblyopia.

In addition to the cholinergic system, other intrinsic neuromodulatory systems that regulate synaptic plasticity, such as dopaminergic and serotonergic systems, have been considered for the treatment of amblyopia. Although both retinal and cortical sites have been suggested^32–39^, the mechanism of action of dopamine in the visual pathway is unknown. Levodopa (an orally administered catecholamine precursor to dopamine) has been evaluated as adjunct amblyopia treatment to occlusion^37,40–46^. Overall improvements in visual acuity were modest and no better than those of occlusion alone, and there were concerns of amblyopia regression after cessation of treatment^37,40–46^.

Serotonergic signaling has been another focus for amblyopia treatment. Administration of fluoxetine, a selective serotonin reuptake inhibitor (SSRI), reinstates ocular dominance plasticity and promotes amblyopia recovery in adult rats^47^. While one clinical trial demonstrated improvement of amblyopia in adolescents and adults using fluoxetine^48^, other clinical trials using SSRIs have not shown a beneficial effect over patching alone^49,50^. and amblyopia regression after treatment cessation was not evaluated. While widely used in children and adults, SSRIs carry concerns for serious adverse effects including suicidal ideation and anxiety^51–53^.

Several studies support the effect of cholinergic potentiation utilizing the AchEI donepezil in perceptual learning^10,12–14^, suggesting that ACh regulates neuroplasticity by targeting the responses of neurons to relevant stimuli^54^. Cholinesterase inhibitor treatment and the resulting increase in ACh promotes cognitive functions such as memory and attention in normal adults^55^, while reduction of cholinergic transmission can prevent experience-dependent changes in fMRI^56^. These results suggest that the cognitive improvements associated with cholinergic enhancement may stem from an augmented capacity and plasticity to learn new information. Rokem and Silver also demonstrated that the beneficial effects on perceptual learning with cholinergic enhancement using donepezil were long lasting and maintained when subjects were tested 5–15 months after training and drug administration were stopped^12^. Identifying the mechanisms of perceptual learning may be of useful importance for the treatment of visual disorders such as amblyopia^57,58^. A pilot study investigated the effect of donepezil on perceptual learning in adults with amblyopia^59^. Nine adult amblyopic patients were trained on low-contrast single-letter identification while treated with 5 mg of daily donepezil for 2 weeks. Although subjects improved with treatment, the improvements were no greater than what was reported by the same authors in six adult amblyopia patients trained on the identical task without the use of donepezil^60^. Still, the two studies are not directly comparable due to small sample sizes and possible confounding factors involving non-randomization and how similarly matched the groups were between the two studies. The lower dosage (5 mg) and shorter treatment time (2 weeks) of donepezil used in the study may have an impact on results. Our study utilized a dose-escalation schedule, and all participants required an increase in their donepezil dose. Of the 5 adult patients in our study, 3 subjects required a dose escalation to 7.5 mg and 2 subjects to 10 mg. The treatment duration for our study was much longer than in previous studies. Subjects improved an average of 0.63 lines during weeks 0–4, 0 lines during weeks 4–8, and 0.58 lines during weeks 8–12 of treatment.

The results of this study suggest that amblyopic eye visual acuity can improve over time with donepezil treatment, and that those gains, while limited to 1.2 lines on average (3.0 lines maximum), are stable after cessation of treatment. Strengths of the present study include a lead-in, 12 weeks of treatment, and wash-out design to include only residual amblyopia and assess durability of gains. Limitations include a small sample size and lack of a placebo-control group. A randomized, controlled trial is needed to determine the efficacy of donepezil as a potential treatment for residual amblyopia. The promising results in the present study support the concept that the critical period of visual cortical plasticity can be pharmacologically manipulated in visually mature humans to treat amblyopia.

## Supplementary Information

Supplementary Figure 1.

## Data Availability

The data that support the findings of this study are available from the corresponding author on reasonable request.

## References

[1] Friedman DS (2009). Prevalence of amblyopia and strabismus in white and African American children aged 6 through 71 months the Baltimore Pediatric Eye Disease Study. Ophthalmology.

[2] Multi-ethnic Pediatric Eye Disease Study (2008). Prevalence of amblyopia and strabismus in African American and Hispanic children ages 6 to 72 months the multi-ethnic pediatric eye disease study. Ophthalmology.

[3] Giordano L (2009). Prevalence of refractive error among preschool children in an urban population: The Baltimore Pediatric Eye Disease Study. Ophthalmology.

[4] Holmes JM, Levi DM (2018). Treatment of amblyopia as a function of age. Vis. Neurosci..

[5] Scheiman MM (2005). Randomized trial of treatment of amblyopia in children aged 7 to 17 years. Arch. Ophthalmol..

[6] Hensch TK, Quinlan EM (2018). Critical periods in amblyopia. Vis. Neurosci..

[7] Bavelier D, Levi DM, Li RW, Dan Y, Hensch TK (2010). Removing brakes on adult brain plasticity: From molecular to behavioral interventions. J. Neurosci..

[8] Takesian AE, Bogart LJ, Lichtman JW, Hensch TK (2018). Inhibitory circuit gating of auditory critical-period plasticity. Nat. Neurosci..

[9] Morishita H, Miwa JM, Heintz N, Hensch TK (2010). Lynx1, a cholinergic brake, limits plasticity in adult visual cortex. Science.

[10] Rokem A, Silver MA (2010). Cholinergic enhancement augments magnitude and specificity of visual perceptual learning in healthy humans. Curr. Biol..

[11] Beer AL, Vartak D, Greenlee MW (2013). Nicotine facilitates memory consolidation in perceptual learning. Neuropharmacology.

[12] Rokem A, Silver MA (2013). The benefits of cholinergic enhancement during perceptual learning are long-lasting. Front. Comput. Neurosci..

[13] Chamoun M (2017). Cholinergic potentiation improves perceptual-cognitive training of healthy young adults in three dimensional multiple object tracking. Front. Hum. Neurosci..

[14] Levi DM, Li RW, Silver MA, Chung STL (2020). Sequential perceptual learning of letter identification and "uncrowding" in normal peripheral vision: Effects of task, training order, and cholinergic enhancement. J. Vis..

[15] Morishita H, Hensch TK (2008). Critical period revisited: Impact on vision. Curr. Opin. Neurobiol..

[16] Holmes JM (2005). A randomized pilot study of near activities versus non-near activities during patching therapy for amblyopia. J. AAPOS.

[17] PEDI Group (2008). A randomized trial of near versus distance activities while patching for amblyopia in children aged 3 to less than 7 years. Opthalmology.

[18] O’Connor AR, Birch EE, Anderson S, Draper H, FR Group (2010). The functional significance of stereopsis. Investig. Ophthalmol. Vis. Sci..

[19] Sahu JK (2013). Effectiveness and safety of donepezil in boys with fragile X syndrome: A double-blind, randomized, controlled pilot study. J. Child Neurol..

[20] Handen BL, Johnson CR, McAuliffe-Bellin S, Murray PJ, Hardan AY (2011). Safety and efficacy of donepezil in children and adolescents with autism: Neuropsychological measures. J. Child Adolesc. Psychopharmacol..

[21] Kishnani PS (2010). Donepezil for treatment of cognitive dysfunction in children with Down syndrome aged 10–17. Am. J. Med. Genet. A.

[22] Kishnani PS (2009). The efficacy, safety, and tolerability of donepezil for the treatment of young adults with Down syndrome. Am. J. Med. Genet. A.

[23] Cubo E (2008). Donepezil use in children and adolescents with tics and attention-deficit/hyperactivity disorder: An 18-week, single-center, dose-escalating, prospective, open-label study. Clin. Ther..

[24] Spiridigliozzi GA (2007). Preliminary study of the safety and efficacy of donepezil hydrochloride in children with Down syndrome: A clinical report series. Am. J. Med. Genet. A.

[25] Doyle RL (2006). Donepezil in the treatment of ADHD-like symptoms in youths with pervasive developmental disorder: A case series. J. Atten. Disord..

[26] Wilens TE (2005). An open trial of adjunctive donepezil in attention-deficit/hyperactivity disorder. J. Child Adolesc. Psychopharmacol..

[27] Heller JH (2004). Donepezil effects on language in children with Down syndrome: Results of the first 22-week pilot clinical trial. Am. J. Med. Genet. A.

[28] Pachaiyappan K, Petti TA, Bangs M, Pfau B, Dumlao S (2003). Urinary incontinence with donepezil treatment in hospitalized children and adolescents with attention deficit hyperactivity disorder. J. Child Adolesc. Psychopharmacol..

[29] Hardan AY, Handen BL (2002). A retrospective open trial of adjunctive donepezil in children and adolescents with autistic disorder. J. Child Adolesc. Psychopharmacol..

[30] Wilens TE, Biederman J, Wong J, Spencer TJ, Prince JB (2000). Adjunctive donepezil in attention deficit hyperactivity disorder youth: Case series. J. Child Adolesc. Psychopharmacol..

[31] Hoopes SP (1999). Donepezil for Tourette's disorder and ADHD. J. Clin. Psychopharmacol..

[32] Djamgoz MB, Wagner HJ (1992). Localization and function of dopamine in the adult vertebrate retina. Neurochem. Int..

[33] Iuvone PM, Tigges M, Fernandes A, Tigges J (1989). Dopamine synthesis and metabolism in rhesus monkey retina: Development, aging, and the effects of monocular visual deprivation. Vis. Neurosci..

[34] Dyer RS, Howell WE, MacPhail RC (1981). Dopamine depletion slows retinal transmission. Exp. Neurol..

[35] Brandies R, Yehuda S (2008). The possible role of retinal dopaminergic system in visual performance. Neurosci. Biobehav. Rev..

[36] Gottlob I, Charlier J, Reinecke RD (1992). Visual acuities and scotomas after one week levodopa administration in human amblyopia. Investig. Ophthalmol. Vis. Sci..

[37] Leguire LE, Walson PD, Rogers GL, Bremer DL, McGregor ML (1995). Levodopa/carbidopa treatment for amblyopia in older children. J. Pediatr. Ophthalmol. Strabismus.

[38] Algaze A (2005). The effects of L-dopa on the functional magnetic resonance imaging response of patients with amblyopia: A pilot study. J. AAPOS.

[39] Yang CI (2003). Functional MRI of amblyopia before and after levodopa. Neurosci. Lett..

[40] Bhartiya P, Sharma P, Biswas NR, Tandon R, Khokhar SK (2002). Levodopa-carbidopa with occlusion in older children with amblyopia. J. AAPOS.

[41] Chatzistefanou KI, Mills MD (2000). The role of drug treatment in children with strabismus and amblyopia. Paediatr. Drugs.

[42] Leguire LE, Rogers GL, Walson PD, Bremer DL, McGregor ML (1998). Occlusion and levodopa-carbidopa treatment for childhood amblyopia. J. AAPOS.

[43] Leguire LE, Walson PD, Rogers GL, Bremer DL, McGregor ML (1993). Longitudinal study of levodopa/carbidopa for childhood amblyopia. J. Pediatr. Ophthalmol. Strabismus.

[44] Mohan K, Dhankar V, Sharma A (2001). Visual acuities after levodopa administration in amblyopia. J. Pediatr. Ophthalmol. Strabismus.

[45] Procianoy E, Fuchs FD, Procianoy L, Procianoy F (1999). The effect of increasing doses of levodopa on children with strabismic amblyopia. J. AAPOS.

[46] Repka MX (2010). Pilot study of levodopa dose as treatment for residual amblyopia in children aged 8 years to younger than 18 years. Arch. Ophthalmol..

[47] Maya Vetencourt JF (2008). The antidepressant fluoxetine restores plasticity in the adult visual cortex. Science.

[48] Sharif MH, Talebnejad MR, Rastegar K, Khalili MR, Nowroozzadeh MH (2019). Oral fluoxetine in the management of amblyopic patients aged between 10 and 40 years old: A randomized clinical trial. Eye (Lond).

[49] Huttunen HJ (2018). Fluoxetine does not enhance the effect of perceptual learning on visual function in adults with amblyopia. Sci. Rep..

[50] Lagas AK, Black JM, Russell BR, Kydd RR, Thompson B (2019). The effect of combined patching and citalopram on visual acuity in adults with amblyopia: A randomized, crossover, placebo-controlled trial. Neural Plast..

[51] Nobile B (2019). Polymorphism A118G of opioid receptor mu 1 (OPRM1) is associated with emergence of suicidal ideation at antidepressant onset in a large naturalistic cohort of depressed outpatients. Sci. Rep..

[52] Ferguson JM (2001). SSRI antidepressant medications: Adverse effects and tolerability. Prim. Care Companion J. Clin. Psychiatry.

[53] Ho D (2012). Antidepressants and the FDA's black-box warning: Determining a rational public policy in the absence of sufficient evidence. Virtual Mentor.

[54] Sarter M, Hasselmo ME, Bruno JP, Givens B (2005). Unraveling the attentional functions of cortical cholinergic inputs: Interactions between signal-driven and cognitive modulation of signal detection. Brain Res. Rev..

[55] Bentley P, Husain M, Dolan RJ (2004). Effects of cholinergic enhancement on visual stimulation, spatial attention, and spatial working memory. Neuron.

[56] Thiel CM, Friston KJ, Dolan RJ (2002). Cholinergic modulation of experience-dependent plasticity in human auditory cortex. Neuron.

[57] Levi DM, Li RW (2009). Perceptual learning as a potential treatment for amblyopia: A mini-review. Vis. Res..

[58] Tsirlin I, Colpa L, Goltz HC, Wong AM (2015). Behavioral training as new treatment for adult amblyopia: A meta-analysis and systematic review. Investig. Ophthalmol. Vis. Sci..

[59] Chung STL, Li RW, Silver MA, Levi DM (2017). Donepezil does not enhance perceptual learning in adults with amblyopia: A pilot study. Front. Neurosci..

[60] Chung ST, Li RW, Levi DM (2012). Learning to identify near-acuity letters, either with or without flankers, results in improved letter size and spacing limits in adults with amblyopia. PLoS One.

