# Supplementary material for: Durable recovery from amblyopia with donepezil
Source: Sci Rep. 2023 Jun 22;13:10161. doi: 10.1038/s41598-023-34891-5 (PMC10287641; doi:10.1038/s41598-023-34891-5)
Supplement: Supplementary file 1 — Supplementary Figure 1. [file 41598_2023_34891_MOESM1_ESM.docx]

**Supplemental Figure 1. Stereoacuity for Individual Subjects**

**

**

Stereoacuity (logarcsec) for individual subjects at each visit.
